# Targeting TRAF3IP2 disrupts cellular energetics through inhibition of NAMPT in triple negative breast cancer

**DOI:** 10.1038/s41598-025-29057-4

**Published:** 2025-12-23

**Authors:** Kurtis Willingham, Amin Izadpanah, Yasmine Rashad, Antonia Reilich, Fatemeh Daneshimehr, Steven Braun, Eckhard U. Alt, Reza Izadpanah

**Affiliations:** 1 Applied Stem Cell Laboratory, Medicine/Section Cardiology, Tulane University School of Medicine, Tulane University, New Orleans, LA USA; 2 Department of Surgery, Tulane University School of Medicine, New Orleans, LA USA; 3 Department of Medicine, Tulane University Health Sciences Center, 1430 Tulane Avenue, New Orleans, LA 70112 USA

**Keywords:** TRAF3IP2, Triple negative breast cancer, NAMPT, NAD, SIRT1, mTOR, ROS, Tumor microenvironment, Metabolism, Cell proliferation, Apoptosis, Cancer, Breast cancer

## Abstract

Triple-negative breast cancer (TNBC) is characterized by extensive metabolic alterations that enable its sustained growth therapeutic resistance. Nicotinamide phosphoribyltransferase (NAMPT) catalyzes the first and rate-limiting step in the nicotinamide dinucleotide (NAD) salvage pathway. Elevated NAMPT is associated with increased aggressiveness and poor prognosis in multiple cancers. Previously, we showed the role of TRAF3IP2 in TNBC tumorigenesis. Here, we aim to show that the anti-tumorigenic effects resulting from TRAF3IP2 inhibition are driven in part by decreases in cellular energetics in TNBC cells. Results show that inhibition of TRAF3IP2 leads to significant decrease in NAMPT expression, reduced NAD and ATP production, and disruption of TNBC bioenergetics through cell line-specific alterations in glycolysis and mitochondrial function. Notably, the established MDA-MB-231 line and the patient-derived 4IC model exhibited distinct OCR responses, underscoring metabolic heterogeneity across TNBC models. Additionally, this study showed that targeting TRAF3IP2 disrupts cellular energetics by affecting AMPK/LKB1 and mTOR signaling pathways and increasing reactive oxygen species (ROS) levels, ultimately leading to reduced cell viability and increased apoptosis. These findings suggest that TRAF3IP2 plays a critical role in maintaining TNBC bioenergetics and represents a potential target for therapeutic intervention.

**Supplementary Information:**

The online version contains supplementary material available at 10.1038/s41598-025-29057-4.

## Introduction

Breast cancer has become the most diagnosed malignancy worldwide, with an increasing incidence rate, currently representing 11.7% of all newly diagnosed cancers^1^. Triple-negative breast (TNBC) accounts for approximately 15–20% of all breast cancers and is characterized as a highly aggressive malignancy with poor prognosis and limited treatment options, and reduced overall survival, due to the absence of estrogen receptor, progesterone receptor, and HER2 expression^2,3^. TNBC is characterized by reliance on bioenergetic pathways due to metabolic reprogramming. Understanding these metabolic dependencies is crucial for identifying new therapeutic targets. Cancer, including TNBC, often exhibits altered metabolism to support rapid growth and survival. One key aspect of this metabolic reprogramming is the increased reliance on glycolysis and mitochondrial oxidative phosphorylation for ATP production^4,5^. Nicotinamide phosphoribosyltransferase (NAMPT) and Sirtuin 1 (SIRT1) are critical regulators of cellular metabolism. NAMPT is involved in the NAD + salvage pathway, which is essential for maintaining cellular NAD + levels, while SIRT1 is a NAD dependent deacetylase that regulates various metabolic processes and stress responses.

TRAF3IP2 (TRAF3 Interacting Protein 2) has been implicated in various cellular processes, including inflammation, immunity, and tumorigenesis. Previously, we showed the role of TRAF3IP2 in TNBC and glioblastoma tumorigenesis, and showed that the inhibition of TRAF3IP2 prevents tumor growth and cancer cell proliferation in vitro and in vivo^6–8^. Previous research has shown that TRAF3IP2 regulates key signaling pathways, including NF-κB and JNK, which are involved in cellular stress responses and survival^9^.

This study aims to elucidate the potential link between TRAF3IP2 and metabolic regulation in cancer cells. We hypothesize that inhibiting TRAF3IP2 disrupts TNBC cell metabolism, leading to decreased ATP production, impaired mitochondrial function, and reduced cell viability.

## Methods

### Database analysis

Protein expression data from the Clinical Proteomic Tumor Analysis Consortium (CPTAC) was analyzed and visualized from The University of Alabama at Birmingham Cancer data analysis Portal (https://ualcan.path.uab.edu/) and Breast Cancer Gene-Expression Miner v5.2 (bc-GenExMiner v5.2) (https://bcgenex.ico.unicancer.fr)^10,11^. Data is visualized as Z-value, representing standard deviations from median across samples within cancer subtype samples. Samples were normalized by Log_2_ spectral count ratio values and normalized within samples, then across samples.

### Cell-culture and reagents

MDA-MB-231 (MDA) was purchased from ATCC (Rockville, MD) and cultured in DMEM media (cat# 11965092, Thermo Fisher Scientific, Waltham, MA) supplemented with 10% fetal bovine serum (Peak Serum Inc., Wellington Colorado) and 1% penicillin-streptomycin (Thermo Fisher Scientific, Waltham, MA). TU-BcX-4IC (4IC), a patient-derived xenograft TNBC cell line, was graciously provided by Matthew Burow, PhD, and were cultured in DMEM supplemented with 10% fetal bovine serum, 1% antibiotic-antimycotic (cat# 15240112), MEM amino acid solution (cat# 11130051), non-essential amino acid solution (cat# 11140050), sodium pyruvate (cat# 11360070), and insulin (cat# 12585014) acquired from Gibco, Inc. (Billings, MO). Adipose-derived stem cells (ASC) were isolated from adipose tissue, dissociated using Matrase enzymatic reagent and separated using the Transpose RT Processing Unit (Ingeneron, Houston TX). Isolated ASC cells were cultured in MEM media (cat# 11095080, Thermo Fisher Scientific, Waltham, MA) supplemented with 20% fetal bovine serum and 1% antibiotic/antimycotic. TRAF3IP2 expression was silenced according to the previous reports^6–8^. Briefly, cells were transduced using lentivirus containing a puromycin resistance gene and either a sequence coding for shRNA TRAF3IP2 (TRAF3IP2KD) or a nonsense scrambled vector (SCR). Transduced cells were selected and labeled depending on their lentiviral transduction type (MDA_TRAF3IP2KD_, MDA_SCR_; 4IC_TRAF3IP2KD_, 4IC_SCR_). Cells were incubated at 37° Celsius at 5% CO_2_. NAMPT inhibitor, FK866 was purchased from Selleckchem chemicals (cat#S2799, Houston, TX).

### Quantitative real-time PCR (qPCR)

RNA was isolated using RNeasy Micro Kit (cat# 74004, Qiagen, Hilden, Germany), and transcribed into complimentary DNA using iScript cDNA Synthesis Kit (cat# 1708891, Bio-Rad Laboratories, Hercules, CA). mRNA levels were quantified using 10 ng cDNA, iTaq universal SYBR Green Supermix (cat# 1725124, Bio-Rad Laboratories, Hercules, CA). Fold change was assessed using the ΔΔCT method, with β-actin serving as a housekeeping gene for normalization. The TRAF3IP2 primer used is a proprietary sequence. The primers used are listed in Table 1.

**Table 1:** List of primers.

Primer targets	Forward primer	Reverse primer
NAMPT	5’AGTGGTGCCTGTGTATT-3’	5’TGCCTGTATCTGTGGTCAG-3’
SIRT1	5’AAGGAAAACTACTTCGCAAC-3’	5’GGAACCATGACACTGAATTATC-3’
BIRC5	5’TGTCTCCTCATCCACCTGAA-3’	5’TCCCTGGCTCCTCTACTGTT-3’
mTOR	5’GGAGGAGAAATTTGATCAGG-3’	5’GGGCAACAAATTAAGGATTG-3’
RPTOR	5’CGGAGTTTCCTTTAACAGTG-3’	5’CTGTTGAGTACTTTCATGGC-3’
RICTOR	5’AAATGCATGAAGAAGCAGAG-3’	5’AACAGTGTACAGAAGATACTCC-3’
β-actin	5′CTGGAACGGTGAAGGTGA-3′	5′AAGGGACTTCCTGTAACA-3′

### NAD quantification

NAD levels were quantified using an NAD/NADH quantitation kit (cat# MAK037-1KT; MilliporeSigma, Rockville, MD). Equal quantity of MDA cells (2 × 10^5^) was harvested from each treatment group, cultured with either FK866 (10nM) or vehicle (DMSO) (SCR, TRAF3IP2KD, SCR + FK866, TRAF3IP2KD + FK866). NAD was isolated and quantified according to the NAD/NADH kit’s colorimetric assay protocol, and the percentage of relative NAD concentration in each group was normalized to the SCR vehicle treated group. Optical density was measured using BMG Labtech’s FLUOstar Optima multi-detection microplate reader (BMG Labtech, Cary, NC). Values were normalized to total protein and expressed relative to DMSO-treated controls.

### Extracellular flux assay

Live metabolic activity was assessed using the Agilent Seahorse XFe24 Analyzer (Agilent Technologies, Santa Clara, CA). Glycolysis was assessed with the Seahorse XF Glycolytic Rate Assay Kit Cells (cat# 103344-100; Agilent Technologies, Santa Clara, CA), and mitochondrial stress was assessed using the Seahorse XF Mito Stress Test Kit (cat# 103015-100; Agilent Technologies, Santa Clara, CA). Cells were seeded overnight prior in a confluent monolayer. After analysis, wells were normalized by dividing oxygen consumption rate (OCR) for Mito Stress Kit and proton efflux rate (PER) for Glycolytic Rate Assay by ug protein, measured using BCA protein assay (cat# 23225; Thermo Scientific, Waltham, MA).

### ROS detection

Treatment and control cells were seeded overnight in equal quantities in a 96 well plate. ROS production was assessed using a ROS/Superoxide Detection Assay Kit (cat# ab139476, Abcam, Cambridge, UK), following protocol for incubation of treatment types and positive control (SCR + pyocyanin, TRAF3IP2KD + pyocyanin). Plates were then analyzed using BMG Labtech’s FLUOstar Optima multi-detection microplate reader (BMG Labtech, Cary, NC).

### Antibodies

Protein expression in western blot and immunohistochemistry (IHC) samples were analyzed using the antibodies listed in Table 2:

**Table 2:** List of antibodies.

Antibody	Catalog number	Vendor
TRAF3IP2	a6776	Abclonal, Woburn, MA
Sirt1	A17307	Abclonal, Woburn, MA
AMPKa1/AMPKa2	A17290	Abclonal, Woburn, MA
Phospho-AMPK (p-AMPK)	AP0116	Abclonal, Woburn, MA
LKB1	A2122	Abclonal, Woburn, MA
Phospho-LKB1 (Phospho-STK11-S428)	AP1108	Abclonal, Woburn, MA
Survivin	A1551	Abclonal, Woburn, MA
Caspase-8	A0215	Abclonal, Woburn, MA
β-actin	AC004	Abclonal, Woburn, MA
Caspase-3	Ab32351	Abcam, Cambridge, UK
Cleaved Caspase-3	5A1E	Cell Signaling Technology, Danvers MA
Ribosomal protein S6	2217	Cell Signaling Technology, Danvers MA
Phospho-Ribosomal protein S6	4858	Cell Signaling Technology, Danvers MA
Proteinkinase Ca	2056	Cell Signaling Technology, Danvers MA
Phospho-Proteinkinase Ca	9375	Cell Signaling Technology, Danvers MA

### In vivo experiments

Ethical considerations: All in vivo experiments were performed in accordance with protocols approved by the Tulane University Institutional Animal Care and Use Committee (IACUC) and adhered to the ARRIVE guidelines. Mice were anesthetized with isoflurane (2–3% in oxygen) prior to all survival procedures, including flank injections. At study endpoint, euthanasia was conducted following the approved IACUC protocol, which involved CO₂ inhalation followed by cervical dislocation.

Tumors were induced based on previously reported^8^. Immunodeficient NSG mice (NOD scid gamma, strain# 005557; The Jackson Laboratory) were injected in the flank with 1 × 10^6^ cells (in Matrigel) of either 4IC_SCR_ or 4IC_TRAF3IP2KD_ cells (*n* = 5/group) following anesthesia with isoflurane as described above. The 4IC_SCR_ cells generated large tumors (average tumor weight: 3.8 g), the 4IC_TRAF3IP2KD_ cells induced a significantly small tumor at 8 weeks post induction. Animals were euthanized according to approved IACUC procedures, which included CO₂ inhalation followed by cervical dislocation. Tumors were subsequently extracted and analyzed for histological evaluation.

### Western blot analysis

Protein was collected from cell cultures using Mammalian Protein Extraction Reagent (Cat# 78503, Thermo Fisher Scientific, Waltham, MA) supplemented Proteinase Inhibitor Cocktail (Cat# P8340, Sigma-Aldrich, St. Louis, MO). Equal quantities of protein form SCR and TRAF3IP2KD groups were added to wells in a Mini-Protean TGX Precast Protein Gels (Bio-Rad Laboratories, Hercules, CA) and transferred to PVDF membrane (cat# #1620177, Bio-Rad Laboratories, Hercules, CA). Membranes were blocked with 5% w/v concentration of either Bovine Serum Albumin (cat# A7906-50G, Thermo Fisher Scientific, Waltham, MA) or nonfat dry milk (cat# 1706404, Bio-Rad Laboratories, Hercules, CA) for one hour and incubated overnight at 4 °C with primary antibodies. Afterwards, membranes were then incubated with secondary antibodies for one hour at room temperature, then incubated in Clarity Western ECL Substrate (cat# 1705060 Bio-Rad Laboratories, Hercules, CA) for ten minutes. Protein bands were imaged using the Chemidoc imaging system from Bio-Rad Laboratories. Relative intensity was assessed using Imagej^12^, with antibody intensity normalized to β-actin intensity.

### Flow cytometry

MDA_SCR_, MDA_TRAF3IP2KD_, MDA_SCR+FK866_, and MDA_TRAF3IP2KD+FK866_ cells (5 × 10^5^) were incubated with APC-labeled annexin-v (5 µl) and propidium iodide (PI) (5 µl) for 10 min. Stained cells were analyzed using the BD FACSMelody™ Cell Sorter (BD Biosciences, Franklin Lakes, NJ). 5000 cells were analyzed for each treatment type. Quantification of cells positive and/or negative for annexin-v and PI was assessed and visualized using Kaluza software (Beckman Coulter, Brea, CA; https://www.beckman.com/flow-cytometry/software/kaluza). Cell incubation, flow cytometry, software analysis, and visualization were completed by the Flow Cytometry and Cell Sorting Core at Tulane University (New Orleans, LA).

### Statistical analysis

Statistics were performed between treatment types with at least three samples (N > = 3). Data is expressed through mean ± standard deviation (S.D.). Outliers were defined by samples greater than or less than the mean multiplied by 2 times S.D. Unpaired Student T-test was used to assess significance between two groups, with *p* < 0.05 considered statistically significant. Statistics were measured and figures generated using Graphpad Prism 10 (Graphpad Software, Boston, MA).

### Data availability

All data generated or analyzed during this study are included in this published article.

## Results

### Elevated TRAF3IP2 expression correlates with poor prognosis in triple-negative breast cancer

**Fig. 1:**
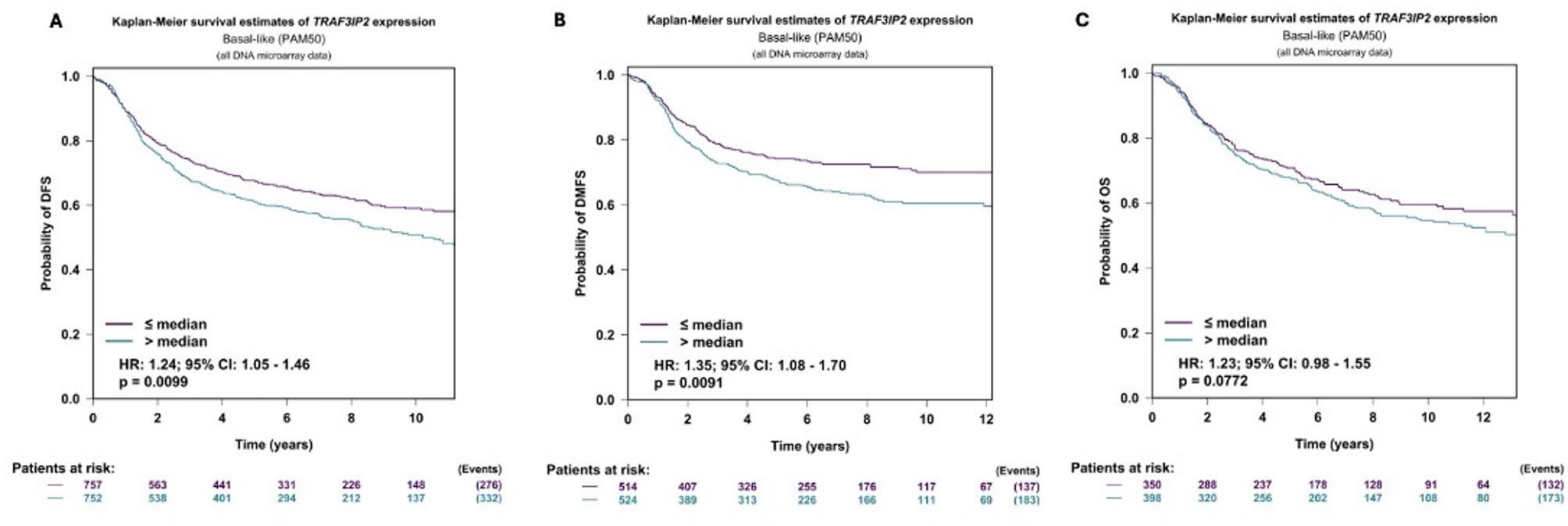
Kaplan-Meier survival analyses of TNBC patients stratified by TRAF3IP2 expression. (**A**) Disease-Free Survival (DFS; HR = 1.24 [95% CI 1.05–1.46], *p* = 0.0099). (**B**) Distant Metastasis-Free Survival (DMFS; HR = 1.35 [95% CI 1.08–1.70], *p* = 0.0091), and (**C**) Overall Survival (OS; HR = 1.23 [95% CI 0.98–1.55], *p* = 0.0772) for basal-like (PAM50) breast cancer cases in Breast Cancer Gene-Expression Miner. Patients were categorized based on median TRAF3IP2 expression (≤ median vs. > median). The x-axis shows time (years), and the y-axis shows the probability of survival. “Patients at risk” is shown below each plot in matching curve colors.

To further investigate the clinical relevance of TRAF3IP2 in TNBC, we examined publicly available data from Breast Cancer Gene-Expression Miner v5.2 (bc-GenExMiner v5.2), focusing on basal-like (PAM50) breast cancer cases. Kaplan-Meier survival curves revealed that patients with higher TRAF3IP2 expression exhibited significantly worse outcomes. As shown if Fig. 1A–C, elevated TRAF3IP2 correlated with reduced disease-free survival (DFS) (HR = 1.24, 95% CI: 1.05–1.46; *p* = 0.0099) (Fig. 1A). Data also showed that high TRAF3IP2 expression was associated with lower distant metastasis-free survival (DMFS) (HR = 1.08, 95% CI: 1.08–1.70; *p* = 0.0091) (Fig. 1B) and overall survival (OS) (Fig. 1C) indicating that patients with elevated TRAF3IP2 expression experienced poorer long-term survival.

### TRAF3IP2 inhibition reduces the elevated levels of NAMPT and SIRT1 expression in TNBC cells

**Fig. 2:**
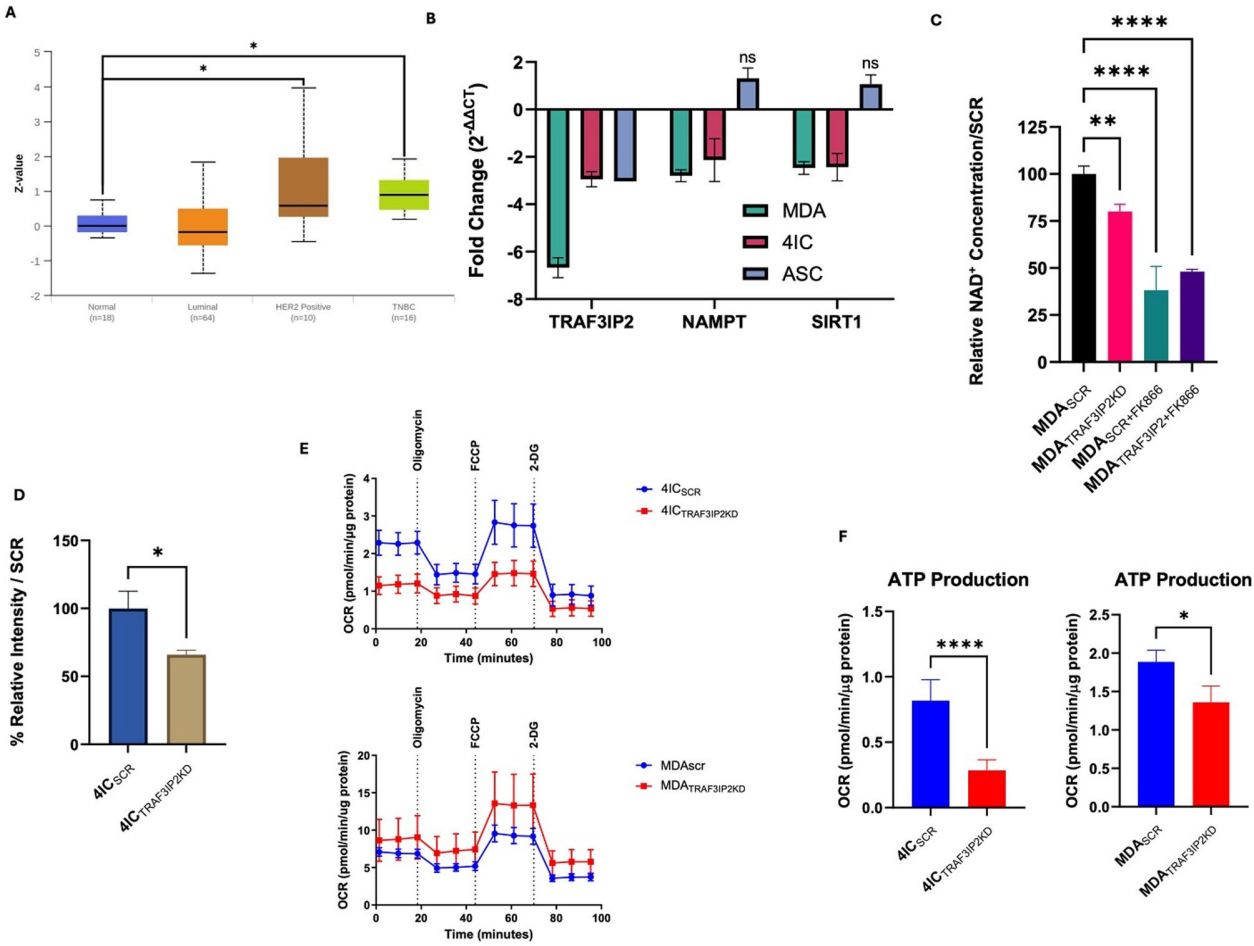
Inhibition of TRAF3IP2 reduces NAD associated expression in TNBC cells. Analysis of TNBC samples from CPTAC shows that NAMPT protein expression is increased in TNBC samples, compared to normal breast tissue samples (**A**). qPCR analysis demonstrated that knockdown of TRAF3IP2 (TRAF3IP2KD) significantly reduces NAMPT and SIRT1 mRNA levels in TNBC samples (MDA, 4IC) (all samples ≤ 0.05), with no change in non-malignant control cells (ASC). The bar graph shows the effect of silencing TRAF3IP2 in each cell line compared to their corresponding controls (SCR-transduced cells) for each cell types of MDA, 4IC, and ASCs (**B**). TRAF3IP2KD leads to a significant decrease in available NAD in MDA cells, compared to SCR controls (**C**). Quantification of ATP/ADP ratio in 4IC_TRAF3IP2KD_ cells shows a significant decline in the ratio of the quantity of ATP to ADP, compared to the 4IC_SCR_ controls (**D**). OCR was measured before and after sequential injections of oligomycin, FCCP, and rotenone/antimycin A. ATP-linked respiration was calculated as basal OCR minus post-oligomycin OCR. Seahorse profiles illustrate a pronounced reduction in OCR for TRAF3IP2KD cells compared to their SCR counterparts (**E**). A summary of basal and maximal OCR values (**F**) confirms diminished mitochondrial function upon TRAF3IP2 knockdown. *P < 0.05; **P < 0.01; ***P < 0.005; ****P < 0.001; n/s = non-significant.

Analysis was conducted to assess whether NAMPT expression is elevated in subtype-specific manner. Data from CPTAC database reveals that among breast cancer subtypes, TNBC tumors show a significant increase in NAMPT expression compared to normal breast samples (Fig. 2A). This implicates NAMPT involvement with increased NAD development, leading to TNBC-associated phenotype of tumor growth and treatment resistance.

To assess the effects of TRAF3IP2 on the TNBC metabolism, we utilized lentiviral vector containing silencer for TRAF3IP2^8^ to suppress its expression in MDA-MB231 (MDA) cells and TU-BcX-4IC (4IC) cells, which are patient-derived metaplastic TNBC cells^13^. The successful knockdown of TRAF3IP2 expression in these cells was validated by qRT-PCR and western blotting. Concurrent with TRAF3IP2 inhibition, our results demonstrated a reduction in NAMPT and SIRT1 expression in both MDA and 4IC cells. Adipose tissue derived stem cells (ASCs) were used as non-malignant cell controls in these experiments. ASCs with TRAF3IP2 knockdown (ASC_TRAF3IP2KD_) did not exhibit changes in TRAF3IP2, NAMPT or SIRT1 levels, indicating a cancer-specific effect of TRAF3IP2 silencing (Fig. 2B).

### NAMPT decline leads to reduced NAD and ATP production

To investigate the relationship between TRAF3IP2, NAMPT levels, and NAD availability, we utilized a colorimetric assay to quantify NAD levels in TRAF3IP2KD cells, with or without FK866, a NAMPT inhibitor. The results demonstrated that TRAF3IP2 silencing significantly reduced NAD levels, and this reduction was further enhanced in TRAF3IP2KD cells treated with FK866 (Fig. 2C). We then examined the effect of TRAF3IP2 silencing on ATP production levels. Since NAD is crucial for ATP production, we assessed ATP levels using a bioluminescence assay and Agilent Seahorse mitochondrial stress assay. TRAF3IP2KD cells exhibited a decreased ATP/ADP ratio (Fig. 2D). Additionally, Seahorse analysis revealed that although MDA_TRAF3IP2KD_ cells exhibit a slight rise in basal oxygen consumption rate (OCR), the calculated ATP-linked respiration is significantly reduced, paralleling the decrease observed in 4IC cells, thereby confirming impaired mitochondrial ATP production upon TRAF3IP2 knockdown (Fig. 2E). Consistent with these findings, we observed a significant reduction in total ATP production in both MDA and 4IC cells (Fig. 2F).

**Fig. 3:**
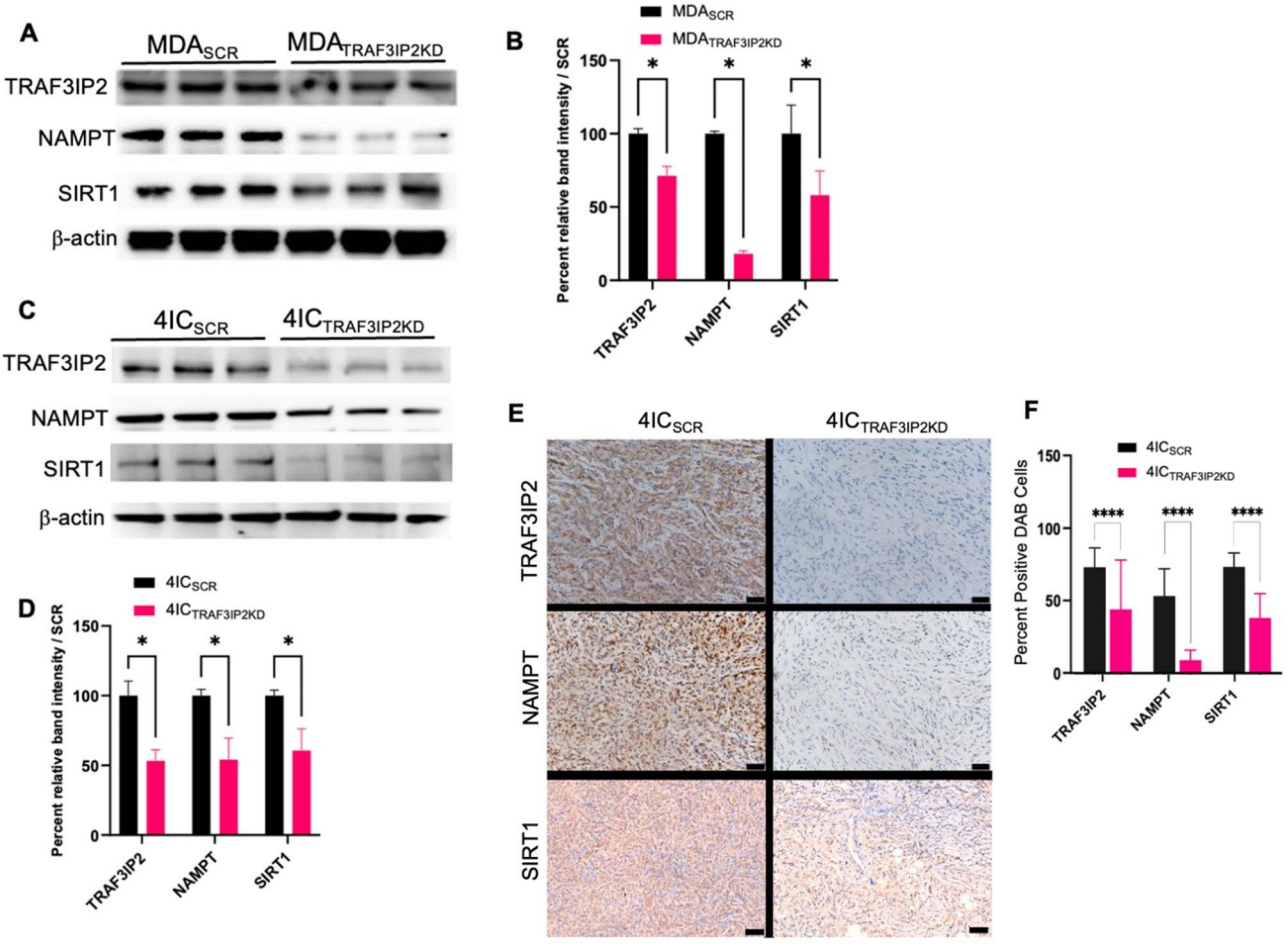
Protein analysis shows that knockdown of TRAF3IP2 alters protein levels of NAD salvage pathway markers in TNBC. Western blot analysis shows that TRAF3IP2KD results in a significant decrease of NAMPT and SIRT1 expression in MDA_TRAF3IP2KD_ (**A**, **B**) and 4IC_TRAF3IPSKD_ (**C**, **D**), compared to controls (MDA_SCR_, 4IC_SCR_) *P < 0.05. Representative Immunohistochemical analysis of TRAF3IP2 (top), NAMPT (middle), and SIRT1 (bottom) in 4IC_SCR_ (left) and 4IC_TRAF3IP2KD_ (right) xenograft tumors; scale bar = 100 μm (**E**). Quantitative analysis showing percent DAB-positive tumor cells for TRAF3IP2, NAMPT, and SIRT1 (mean ± SD; ****P < 0.0001) (**F**).

Further analysis showed that targeting TRAF3IP2 markedly reduced TRAF3IP2, NAMPT, and SIRT1 protein expression in MDA-MB-231 cells (Fig. 3A, B) and TU-BcX-4IC cells (Fig. 3C, D). Immunohistochemical analysis confirmed significant decreases in TRAF3IP2, NAMPT, and SIRT1 staining in 4IC_TRAF3IP2KD_ xenografts compared to 4IC_SCR_ control tumors (Fig. 3E). Quantitative DAB H-score analysis (Fig. 3F) demonstrate highly significant reductions in TRAF3IP2 (4IC_SCR_: 75 ± 1% vs. 4IC_TRAF3IP2KD_: 45 ± %), NAMPT (55 ± 1% vs. 10 ± %), and SIRT1 (65 ± % vs. 40 ± %); *p* < 0.0001.

### Silencing TRAF3IP2 disrupts cellular energetics following increasing AMPK and LKB1 phosphorylation

**Fig. 4:**
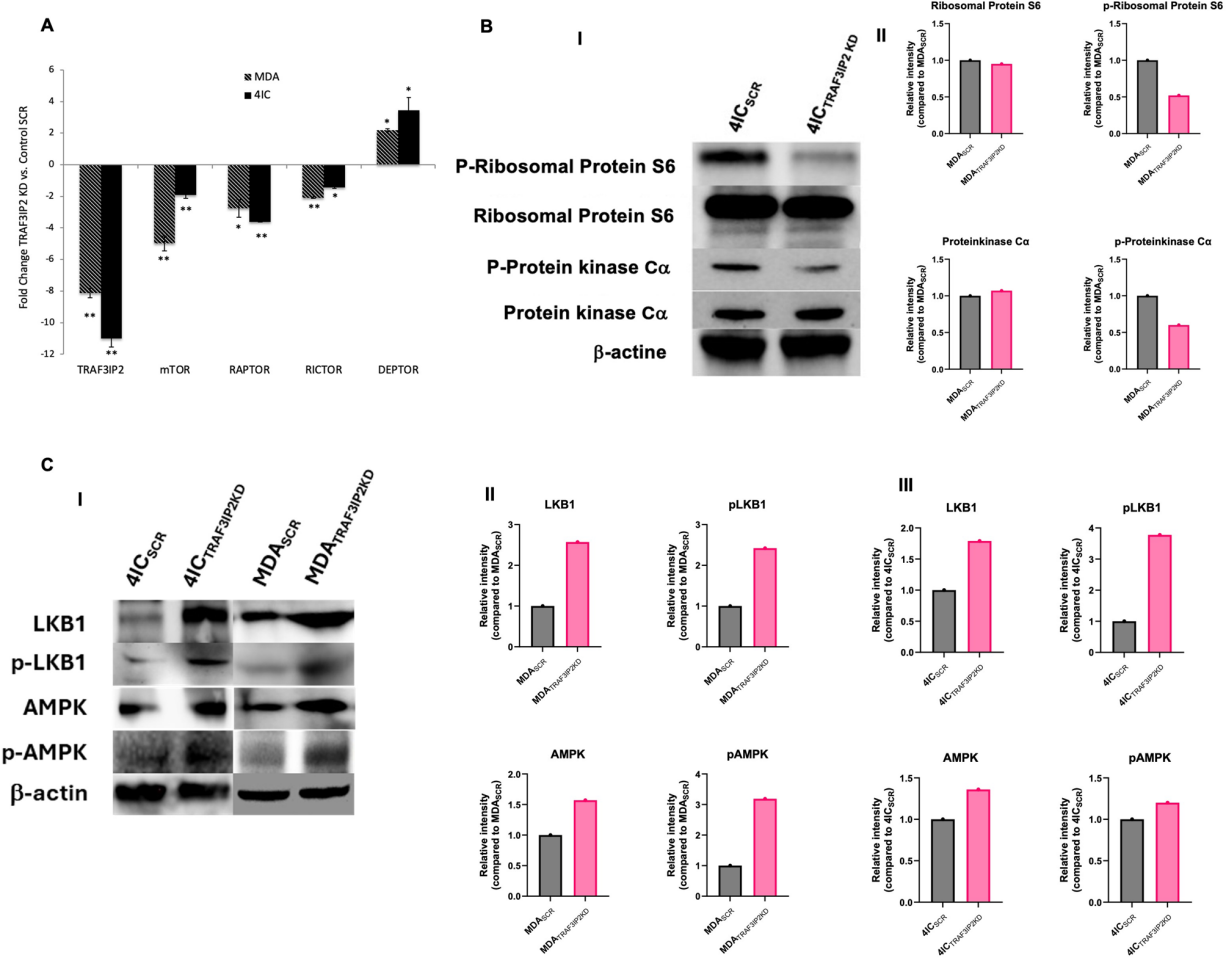
Targeting TRAF3IP2 alters nutrient sensing pathways in TNBC cells. RT-qPCR analysis shows that silencing TRAF3IP2 decreases mTOR, RAPTOR, and RICTOR expression while increasing the mTOR inhibitor DEPTOR (**A**) *P < 0.05; **P < 0.01. Western blot analysis of tumor tissues showed reduced phosphorylation of ribosomal protein S6 and protein kinase Cα in TRAF3IP2-silenced tumors, whereas total protein levels remain largely unchanged (**B-I**), with corresponding densitometric quantification confirming selective decreases in the phosphorylated forms (**B-II**). Western blot analysis shows that TRAF3IP2 silencing increases phosphorylation of AMPK and LKB1 in both 4IC_TRAF3IP2KD_ and MDA_TRAF3IP2KD_, compared to SCR controls (**C-I**), with densitometric quantification presented separately for MDA (**C-II**) and 4IC (**C-III**).

To mechanistically delineate the effects of TRAF3IP2 on TNBC cellular energetics, we first examined transcriptional changes in mTOR-pathway components by RT-qPCR. TRAF3IP2 knockdown decreased expression of mTOR, RAPTOR, and RICTOR, while increasing expression of the mTOR inhibitor DEPTOR (Fig. 4A). We next evaluated downstream markers associated with mTOR signaling in tumor tissue. Western blot analysis showed that TRAF3IP2 silencing markedly reduced phosphorylation of ribosomal protein S6 and protein kinase Cα, whereas total protein levels remained largely unchanged (Fig. 4B-I). Quantitative analysis confirmed these findings, demonstrating significant decreases in the phosphorylated forms without corresponding changes in total protein expression (Fig. 4B-II). Then, we analyzed the impact of TRAF3IP2 on activation of cellular energy-stress sensors, AMPK and Liver Kinase B1 (LKB1). Western blots demonstrated increased phosphorylation of AMPK and LKB1 in both MDA-MB-231 and 4IC cells following TRAF3IP2 knockdown (Fig. 4C-I-III). Together, these transcriptional, translational, and phosphorylation changes highlight a dual effect of TRAF3IP2 silencing: disruption of mTOR complex signaling combined with concomitant activation of the AMPK/LKB1 energy-stress axis. These alterations suggest that TRAF3IP2 inhibition reduces energy availability and compromises metabolic flexibility in TNBC.

### Silencing TRAF3IP2 decreases glycolysis, increases ROS, and decreases cell viability

**Fig. 5:**
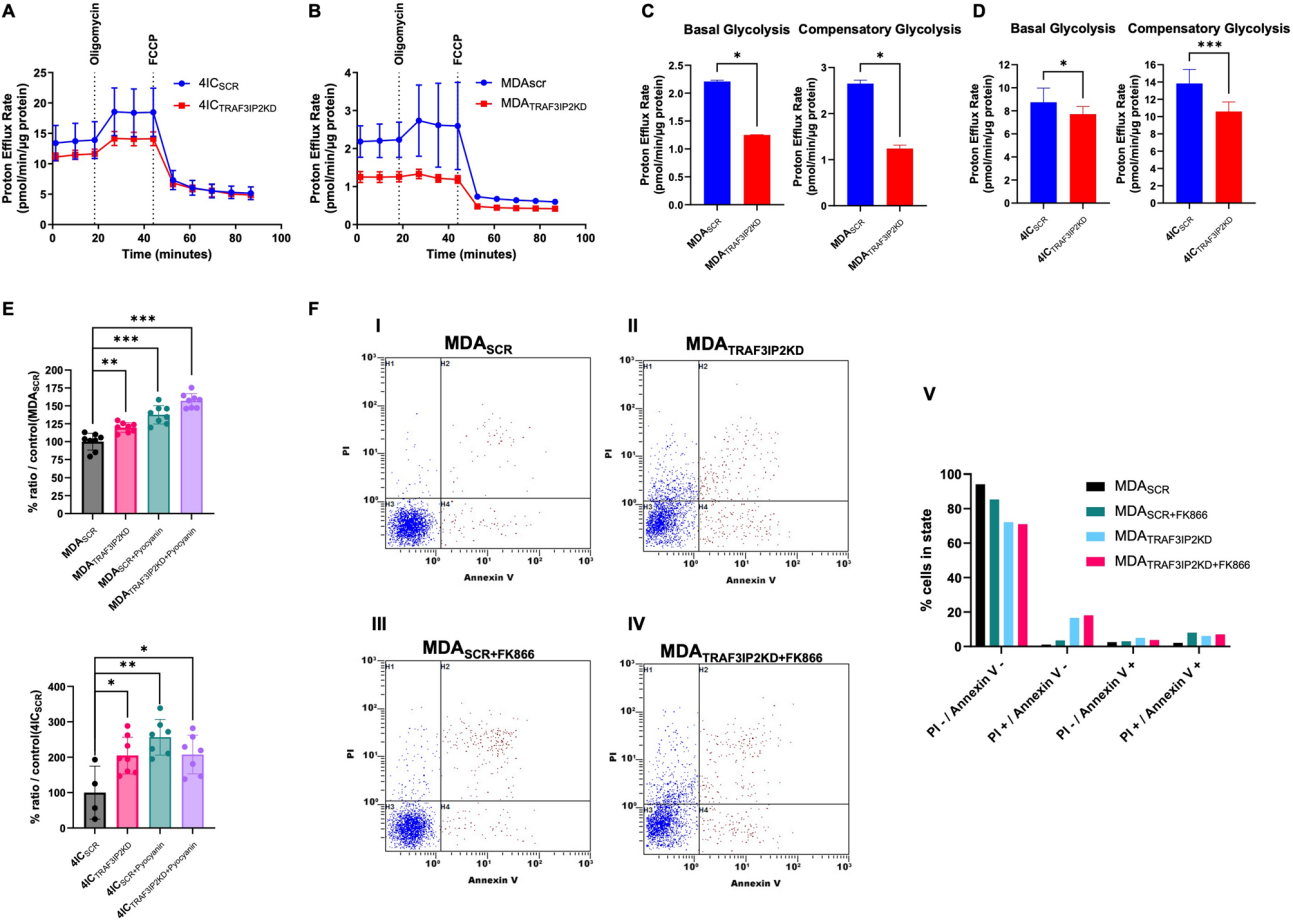
Silencing of TRAF3IP2 decreases glycolysis, increases ROS accumulation and cell death. Glycolysis stress test results TRAF3IP2KD cells compare to control using a Seahorse analyzer. Cells were sequentially treated with glucose (to initiate glycolysis), oligomycin (to shift ATP production toward glycolysis), and 2-deoxyglucose (2‐DG; to inhibit glycolysis). Data are presented as the mean ± SEM of ECAR (mpH/min), with each time point representing an average of replicate wells. Targeting TRAF3IP2 results in decreased basal glycolysis and compensatory glycolytic capabilities in MDA and 4IC cells (**A**–**D**). TRAF3IP2KD is associated with increased ROS in MDA (i) and 4IC (ii) cells, in presence or absence of pyocyanin, an inducer of oxidative stress compared to SCR controls (**E**). Annexin V/PI analysis: representative dot plots of MDA_SCR_, MDA_SCR+FK866_, MDA_TRAF3IP2KD_, and MDA_TRAF3IP2KD+FK866_ cells (left panels) reveals that, compared to controls (I), treatment of NAMPT inhibitor FK866 is associated with a decrease in viable cells (PI - / Annexin V) (II), which further declines following TRAF3IP2KD (III, IV). Bar graph (V) shows percentage of cells in each quadrant (*P* < 0.05) (**F**).

To further investigate the role of TRAF3IP2 in the metabolic regulation of malignant cells, we assessed the level of glycolysis in TNBC cells with TRAF3IP2 silencing. Analysis of extracellular acidification rate (ECAR) revealed that the control group started at a modestly higher baseline, suggesting slightly higher basal glycolysis. Upon addition of glucose and oligomycin, the control group showed a more pronounced increase in ECAR, reflecting a robust ability to upregulate glycolysis when oxidative phosphorylation was inhibited. However, ECAR rose more modestly in the TRAF3IP2KD cells, indicating reduced glycolytic capacity. Treatment with 2-deoxyglucose (2‐DG) lowered ECAR in both groups to near‐baseline levels, confirming that the majority of acidification measured was due to glycolysis (Fig. 5A, B). Graphs in Fig. 5C and D show live proton efflux rate measurements indicating a significant reduction in both basal glycolysis and glycolytic capacity in MDA_TRAF3IP2KD_ and 4IC_TRAF3IP2KD_ cells, correlating with decreased ATP production (Fig. 2D and E). Additionally, TRAF3IP2 silencing in MDA and 4IC cells led to an increased accumulation of reactive oxygen species (ROS) (Fig. 5E), along with reduced levels of NAD and decreased expression of SIRT1 (Fig. 2C). Interestingly, treatment with pyocyanin, an inducer of oxidative stress, did not further elevate ROS levels in TRAF3IP2KD cells, which already exhibited high, and likely maximal, ROS levels. Collectively, these findings indicate that the observed reduction in glycolytic activity, coupled with decreased ATP production and increased ROS, would negatively affect TNBC cell viability. These results were corroborated with Annexin V/PI assays, which demonstrate a significantly greater increase in cell death in TRAF3IP2KD cells compared to NAMPT inhibition alone (Fig. 5F). Additionally, the application of FK866, a NAMPT inhibitor, to TRAF3IP2KD cells did not further reduce cell viability compared to untreated TRAF3IP2KD cells. These results underscore the critical role of TRAF3IP2 in sustaining TNBC cell viability by modulating key metabolic pathways and controlling ROS levels.

## Discussion

Our novel findings underscore the pivotal role of TRAF3IP2 as a critical regulator of bioenergetics in TNBC cells. Our analysis of Breast Cancer Gene-Expression Miner data indicates that higher TRAF3IP2 expression is significantly associated with worse DFS, DMFS, and OS in basal-like TNBC. These results align with our experimental observations that TRAF3IP2 is a critical regulator of metabolic pathways in TNBC cells. By demonstrating a strong correlation between TRAF3IP2 expression and poorer clinical outcomes, we highlight the possibility that TRAF3IP2 contributes to both the aggressive phenotype and reduced survival rates characteristic of this breast cancer subtype (Fig. 1A-C). This study also reveals that silencing TRAF3IP2 triggers a significant downregulation of NAMPT and SIRT1 expression (Figs. 2B and 3A–D), leading to a marked reduction in NAD and ATP production (Fig. 2D, E). It is important to note, however, that ATP production was assessed using OCR-based analyses, which provide an indirect measure of ATP synthesis rather than absolute quantification. Nevertheless, the observed reduction in ATP-linked respiration following TRAF3IP2 silencing strongly supports the interpretation that metabolic energy production is impaired. This metabolic disruption directly impairs glycolysis (Fig. 5A, B), effectively depriving TNBC cells of the energy necessary for tumor growth. Moreover, the metabolic stress induced by TRAF3IP2 inhibition activates key energy stress response pathways, specifically AMPK and LKB1 signaling (Fig. 4A). These pathways, typically attenuated in TNBC cells, are reactivated under conditions of energy deprivation, further accelerating cellular metabolism. By depleting the energy reserves of TNBC cells and reactivating these critical stress response pathways, TRAF3IP2 silencing exerts a potent anti-tumor effect, evidenced by the substantial reduction in ATP production and tumor viability. Interestingly, distinct patterns of oxidative phosphorylation changes were observed between the two TNBC models studied: the established MDA-MB-231 cell line and the patient-derived 4IC cell line. This divergence may reflect the intrinsic metabolic heterogeneity of TNBC subtypes, as well as differences arising from their origins (established versus patient-derived). Previous studies have shown that TNBC cell lines exhibit significant metabolic variability, including differences in glycolytic reliance and mitochondrial plasticity^14^. Accordingly, while both models consistently demonstrated a reduction in NAD and glycolytic activity following TRAF3IP2 inhibition, the specific OCR responses underscore the context-dependent metabolic effects of TRAF3IP2 silencing.

NAMPT and SIRT1 play essential roles in cellular metabolism and stress responses^15,16^. NAMPT is a key enzyme in the NAD + salvage pathway, essential for maintaining cellular NAD + levels^17^. NAD + is a crucial cofactor for various metabolic enzymes and is involved in redox reactions, DNA repair, and signaling^18,19^. Overexpression of NAMPT enhances tumorigenic properties, including increased proliferation, stemness, and epithelial-mesenchymal transition (EMT)^20,21^ largely mediated through activation of SIRT1 and other NAD+-dependent enzymes^18^. High SIRT1 expression is associated with poor prognosis in TNBC, correlating with lymph node metastasis and distant metastatic relapse^22,23^. SIRT1 plays multifaceted role in breast cancer including, its role in cell proliferation and survival, metabolic reprogramming, and drug resistance^24–26^. SIRT1 contributes to breast cancer by promoting several pathways that are crucial for cancer cell survival and proliferation. SIRT1 deacetylates and inactivates p53, thereby promoting cell survival and proliferation. SIRT1 also influences the Warburg effect by inhibiting HIF-1α, which is necessary for the metabolic shift to glycolysis in cancer cells^18^. The reduction in NAMPT and SIRT1 expression following TRAF3IP2 inhibition suggests that TRAF3IP2 is integral to maintaining NAD + homeostasis and SIRT1 activity in TNBC cells (Figs. 2B, C and 3A–F).

In addition, the activation of AMPK and LKB1 signaling following inhibition of TRAF3IP2 indicate the role of TRAF3IP2 in regulating cellular energy balance. AMPK is a central energy sensor that is activated in response to low ATP levels^27^, while LKB1 is a tumor suppressor that activates AMPK in response to metabolic stress^28,29^. The increased phosphorylation of AMPK and LKB1 in TRAF3IP2KD cells indicates an energy stress response due to disrupted cellular energetics (Fig. 4A, B).

More importantly, the disruption of mTOR signaling following TRAF3IP2 inhibition highlights the broad impact on cellular metabolism. In TNBC the LKB1 deficiency promotes tumor growth and metastasis by enhancing glycolysis and activating the mTOR- HIF-1α axis, which supports TNBC cells survival and proliferation under metabolic stress^30,31^. As a key regulator of cell growth, proliferation, and metabolism, mTOR forms two distinct complexes, mTORC1 and mTORC2, which regulate different aspects of cellular metabolism^32,33^. mTORC1 is sensitive to nutrient availability and regulates protein synthesis, autophagy, and lipid metabolism^34^. mTORC2 regulates cytoskeletal organization and cell survival^35,36^. AMPK activation suppresses mTORC1 signaling, thus inhibiting cell proliferation^37,38^. The inhibition of TRAF3IP2 leads to a broad disruption of mTOR signaling pathways, as evidenced by decreased expression of mTOR, RAPTOR, and RICTOR, and an increase in DEPTOR expression. This disruption contributes to the anti-tumorigenic effects observed in TRAF3IP2KD cells. The decreased phosphorylation of protein kinase Ca and ribosomal protein 6 upon targeting TRAF3IP2 highlights a strategic impairment of mTOR pathways crucial for sustaining TNBC cell metabolism. Specifically, reduced mTORC2 activity, indicated by decreased protein kinase Ca phosphorylation, suggests compromised cytoskeletal organization and survival signaling mediated by Rictor. Concurrently, decreased ribosomal protein 6 phosphorylation reflects inhibited mTORC1 activity, suggesting a reduced protein synthesis and cell growth pathways orchestrated by Raptor. Moreover, the increased DEPTOR expression implies augmented negative regulation of mTOR functions (Fig. 4C, D).

Additionally, the reduction in glycolytic function observed in TRAF3IP2KD cells further supports the role of TRAF3IP2 in maintaining metabolic homeostasis in TNBC. Cancer cells often rely on aerobic glycolysis, also known as the Warburg effect, to generate ATP and biosynthetic precursors^39^. The decreased basal glycolysis and glycolytic capacity in TRAF3IP2KD cells indicate that TRAF3IP2 is essential for maintaining glycolytic flux in TNBC cells (Fig. 5A-B). Previously, we reported that silencing TRAF3IP2 resulted in reduced tumor size which is linked to reduced malignant cell proliferation^8^, this can be very well associated with reduced ATP levels as a result of targeting TRAF3IP2.

The increase in ROS and apoptosis upon TRAF3IP2 inhibition (Fig. 5C) further underscores its potential as a therapeutic target. Elevated ROS levels can induce oxidative stress, leading to cell damage and apoptosis^40–42^. Sirt1 expression is implicated in mitigating excess ROS in cancer cells, leading to increased cancer cell signaling, proliferation and resistance to chemotherapy treatments^42^. Inhibition of NAMPT is associated with a decrease in PARP1 expression, leading to increased cell death due to oxidative stress and DNA damage^43^. The increased ROS levels (Fig. 5C) and apoptosis observed in TRAF3IP2KD cells suggest that TRAF3IP2 inhibition disrupts redox homeostasis, contributing to reduced cell viability (Fig. 5D).

### Limitations

While our primary focus in this study was to elucidate the effect of TRAF3IP2 on the metabolic regulation of TNBC cells, there are several noteworthy limitations:

1- Cell Line Selection: We used ASCs as non-malignant controls because they closely represent the stromal and mesenchymal characteristics of the tumor microenvironment. However, we recognize that MCF10A, a commonly used immortalized epithelial cell line, could also serve as a valuable non-malignant control. Future work will include parallel experiments with MCF10A to further validate our findings. 2- Glycolytic Enzyme Analyses: Although our Seahorse XF experiments revealed substantial reductions in glycolysis, the activities and expression levels of key glycolytic enzymes—hexokinase, phosphofructokinase-1, and pyruvate kinase—were not measured. Future investigations will address these enzymes to provide a more comprehensive view of TRAF3IP2’s role in metabolic regulation. 3- Additional Pathway Exploration: While earlier research has shown that TRAF3IP2 influences NF-κB activity, this study concentrated on metabolic regulation via AMPK. The potential effects on MAPK signaling and other proliferation markers will be addressed in subsequent investigations. 4- Mitochondrial and ROS Dynamics: The link between TRAF3IP2 knockdown, mitochondrial changes, and the rise in ROS remains incompletely defined. Further studies are needed to clarify how mitochondrial alterations, and inflammatory responses converge following TRAF3IP2 inhibition. 5- Protein–Protein Interactions: Although we employed FK866 to corroborate our metabolic findings, additional protein–protein interaction studies are essential to fully elucidate the molecular mechanisms by which TRAF3IP2 controls metabolic activity in cancer cells.

## Conclusion

Available data suggest that elevated TRAF3IP2 expression is strongly associated with poor prognosis in TNBC. The results of this study demonstrate that targeting TRAF3IP2 disrupts the NAD pathway by downregulating NAMPT and SIRT1, which in turn leads to reduced NAD and ATP production. This metabolic stress impairs glycolysis, depriving TNBC cells of essential energy resources. Concurrently, TRAF3IP2 knockdown activates the energy-sensing pathways AMPK and LKB1 (normally inactive in TNBC) highlighting the severe energy depletion induced by TRAF3IP2 inhibition. Additionally, the disruption of mTOR signaling, evidenced by decreased mTOR, RAPTOR, and RICTOR expression along with elevated DEPTOR levels, broadly inhibits cellular growth and proliferation. Elevated ROS levels and increased apoptosis further emphasize TRAF3IP2’s critical role in maintaining redox homeostasis. Collectively, these data establish TRAF3IP2 as a key regulator of malignant metabolism and a promising therapeutic target in TNBC.

## Supplementary Information

Below is the link to the electronic supplementary material.

Supplementary Material 1

## Data Availability

All data generated or analyzed during this study are included in this published article.

## Abbreviations

TRAF3IP2: TRAF3 interacting protein 2
NF-kB: Nuclear factor kappa-light-chain-enhancer of activated B cells
NAMPT: Nicotinamide phosphoribosyltransferase
SIRT1: Sirtuin 1
p53: Tumor protein p53
a-p53: Acetylated P53 (K382)
p-p53: Phosphorylated p53 (S392)
LKB1: Liver Kinase B1
p-LKB1: Phosphorylated Liver Kinase B1
AMPK: AMP-activated protein kinase
p-AMPK: Phosphorylated AMP-activated protein kinase
mTOR: Mammalian target of rapamycin
mTORC: Mammalian target of rapamycin complex
RPTOR: Raptor gene
β-actin: Actin beta
ROS: Reactive oxygen species
NAD: Nicotinamide adenine dinucleotide
NMN: Nicotinamide mononucleotide
ADP: Adenosine diphosphate
ATP: Adenosine triphosphate
NMN: Nicotinamide mononucleotide
OCR: Oxygen consumption rate
MDA (MDA-MB231): TNBC cell line
4IC (TU-BcX-4IC): A patient-derived xenograft TNBC cell line
TCGA: The cancer genome atlas
GTeX: Genotype-tissue expression
NSG: NOD scid gamma
DMSO: Dimethyl sulfoxide
DCFH-DA: Dichlorodihydrofluorescein diacetate
PI: Propidium iodide
FK866: NAMPT inhibiting molecule

## References

[1] Sung, H. et al. Global cancer statistics 2020: GLOBOCAN estimates of incidence and mortality worldwide for 36 cancers in 185 countries. *CA Cancer J. Clin.* **71**, 209–249 (2021). 33538338 10.3322/caac.21660

[2] Foulkes, W. D. & Smith, I. E. Reis-Filho, Triple-negative breast cancer. *N Engl. J. Med.* **363**, 1938–1948 (2010). 21067385 10.1056/NEJMra1001389

[3] Bauer, K. R., Brown, M., Cress, R. D., Parise, C. A. & Caggiano, V. Descriptive analysis of Estrogen receptor (ER)-negative, progesterone receptor (PR)-negative, and HER2-negative invasive breast cancer, the so-called triple-negative phenotype: a population-based study from the California cancer registry. *Cancer* **109**, 1721–1728 (2007). 17387718 10.1002/cncr.22618

[4] Yang, Y. et al. NAD + biosynthesis metabolism predicts prognosis and indicates immune microenvironment for breast cancer. *Pathol. Oncol. Res.* **29**, 1610956 (2023). 37006438 10.3389/pore.2023.1610956 PMC10063816

[5] Kim, M. S. et al. Targeting breast cancer metabolism with a novel inhibitor of mitochondrial ATP synthesis. *Oncotarget* **11**, 3863–3885 (2020). 33196708 10.18632/oncotarget.27743 PMC7597410

[6] Izadpanah, A. et al. Targeting TRAF3IP2 inhibits angiogenesis in glioblastoma. *Front. Oncol.* **12**, 893820 (2022). 36046049 10.3389/fonc.2022.893820 PMC9421153

[7] Alt, E. U. et al. TRAF3IP2, a novel therapeutic target in glioblastoma multiforme. *Oncotarget* **9**, 29772–29788 (2018). 30038719 10.18632/oncotarget.25710 PMC6049871

[8] Alt, E. U. et al. Targeting TRAF3IP2, compared to Rab27, is more effective in suppressing the development and metastasis of breast cancer. *Sci. Rep.* **10**, 8834 (2020). 32483202 10.1038/s41598-020-64781-z PMC7264196

[9] Wei, J., Yan, T. & Liang, Y. Targeting TRAF3IP2 alleviates high glucose-induced cardiomyocyte inflammation and apoptosis. *Drug Dev. Res.* **83**, 167–175 (2022). 34260107 10.1002/ddr.21856

[10] Chandrashekar, D. S. et al. A portal for facilitating tumor subgroup gene expression and survival analyses. *Neoplasia* **19**, 649–658 (2017). 28732212 10.1016/j.neo.2017.05.002 PMC5516091

[11] Chandrashekar, D. S. et al. An update to the integrated cancer data analysis platform. *Neoplasia* **25**, 18–27 (2022). 35078134 10.1016/j.neo.2022.01.001 PMC8788199

[12] Schneider, C. A., Rasband, W. S. & Eliceiri, K. W. NIH image to imageJ: 25 years of image analysis. *Nat. Methods*. **9**, 671–675 (2012). 22930834 10.1038/nmeth.2089 PMC5554542

[13] Matossian, M. D. et al. In-depth characterization of a new patient-derived xenograft model for metaplastic breast carcinoma to identify viable biologic targets and patterns of matrix evolution within rare tumor types. *Clin. Transl Oncol.* (2021). 10.1007/s12094-021-02677-8 PMC8732292 34370182

[14] Gong, Y. et al. Metabolic-Pathway-Based subtyping of Triple-Negative breast cancer reveals potential therapeutic targets. *Cell. Metab.* **33**, 51–64e59 (2021). 33181091 10.1016/j.cmet.2020.10.012

[15] Wang, B. et al. NAMPT overexpression in prostate cancer and its contribution to tumor cell survival and stress response. *Oncogene* **30**, 907–921 (2011). 20956937 10.1038/onc.2010.468

[16] Wang, R. H. et al. Hepatic Sirt1 deficiency in mice impairs mTorc2/Akt signaling and results in hyperglycemia, oxidative damage, and insulin resistance. *J. Clin. Invest.* **121**, 4477–4490 (2011). 21965330 10.1172/JCI46243 PMC3204833

[17] Lucena-Cacace, A., Otero-Albiol, D., Jimenez-Garcia, M. P., Munoz-Galvan, S. & Carnero, A. NAMPT is a potent oncogene in colon cancer progression that modulates cancer stem cell properties and resistance to therapy through Sirt1 and PARP. *Clin. Cancer Res.* **24**, 1202–1215 (2018). 29203587 10.1158/1078-0432.CCR-17-2575

[18] Navas, L. E. & Carnero, A. NAD(+) metabolism, stemness, the immune response, and cancer. *Signal. Transduct. Target. Ther.* **6**, 2 (2021). 33384409 10.1038/s41392-020-00354-w PMC7775471

[19] Carreira, A. S. A. et al. Mitochondrial rewiring drives metabolic adaptation to NAD(H) shortage in triple negative breast cancer cells. *Neoplasia* **41**, 100903 (2023). 37148658 10.1016/j.neo.2023.100903 PMC10192916

[20] Amjad, S. et al. Role of NAD(+) in regulating cellular and metabolic signaling pathways. *Mol. Metab.* **49**, 101195 (2021). 33609766 10.1016/j.molmet.2021.101195 PMC7973386

[21] Heske, C. M. Beyond energy metabolism: exploiting the additional roles of NAMPT for cancer therapy. *Front. Oncol.* **9**, 1514 (2019). 32010616 10.3389/fonc.2019.01514 PMC6978772

[22] Wu, M. et al. Expression of SIRT1 is associated with lymph node metastasis and poor prognosis in both operable triple-negative and non-triple-negative breast cancer. *Med. Oncol.* **29**, 3240–3249 (2012). 22661383 10.1007/s12032-012-0260-6

[23] Zhang, H. et al. The prognostic implications of SIRTs expression in breast cancer: a systematic review and meta-analysis. *Discov Oncol.* **13**, 69 (2022). 35927590 10.1007/s12672-022-00529-7 PMC9352848

[24] Varghese, B. et al. SIRT1 activation promotes energy homeostasis and reprograms liver cancer metabolism. *J. Transl Med.* **21**, 627 (2023). 37715252 10.1186/s12967-023-04440-9 PMC10504761

[25] Garcia-Peterson, L. M. & Li, X. Trending topics of SIRT1 in tumorigenicity. *Biochim. Biophys. Acta Gen. Subj.* **1865**, 129952 (2021). 34147543 10.1016/j.bbagen.2021.129952 PMC8277705

[26] Kuo, S. J., Lin, H. Y., Chien, S. Y. & Chen, D. R. SIRT1 suppresses breast cancer growth through downregulation of the Bcl-2 protein. *Oncol. Rep.* **30**, 125–130 (2013). 23673452 10.3892/or.2013.2470

[27] Schuster, S. et al. FK866-induced NAMPT Inhibition activates AMPK and downregulates mTOR signaling in hepatocarcinoma cells. *Biochem. Biophys. Res. Commun.* **458**, 334–340 (2015). 25656579 10.1016/j.bbrc.2015.01.111

[28] Li, N., Huang, D., Lu, N. & Luo, L. Role of the LKB1/AMPK pathway in tumor invasion and metastasis of cancer cells (Review). *Oncol. Rep.* **34**, 2821–2826 (2015). 26398719 10.3892/or.2015.4288

[29] Dupuy, F. et al. LKB1 is a central regulator of tumor initiation and pro-growth metabolism in ErbB2-mediated breast cancer. *Cancer Metab.* **1**, 18 (2013). 24280377 10.1186/2049-3002-1-18 PMC4178213

[30] Zhao, R. X. & Xu, Z. X. Targeting the LKB1 tumor suppressor. *Curr. Drug Targets*. **15**, 32–52 (2014). 24387336 10.2174/1389450114666140106095811 PMC3899349

[31] Fan, D., Ma, C. & Zhang, H. The molecular mechanisms that underlie the tumor suppressor function of LKB1. *Acta Biochim. Biophys. Sin (Shanghai)*. **41**, 97–107 (2009). 19204826 10.1093/abbs/gmn011

[32] Morita, M. et al. mTORC1 controls mitochondrial activity and biogenesis through 4E-BP-dependent translational regulation. *Cell. Metab.* **18**, 698–711 (2013). 24206664 10.1016/j.cmet.2013.10.001

[33] Watanabe, R., Miyata, M. & Oneyama, C. Rictor promotes tumor progression of rapamycin-insensitive triple-negative breast cancer cells. *Biochem. Biophys. Res. Commun.* **531**, 636–642 (2020). 32819718 10.1016/j.bbrc.2020.08.012

[34] Rabanal-Ruiz, Y., Otten, E. G. & Korolchuk, V. I. mTORC1 as the main gateway to autophagy. *Essays Biochem.* **61**, 565–584 (2017). 29233869 10.1042/EBC20170027 PMC5869864

[35] Cordover, E. et al. KPT-9274, an inhibitor of PAK4 and NAMPT, leads to downregulation of mTORC2 in triple negative breast cancer cells. *Chem. Res. Toxicol.* **33**, 482–491 (2020). 31876149 10.1021/acs.chemrestox.9b00376 PMC9316853

[36] Sun, Y. et al. mTORC2: a multifaceted regulator of autophagy. *Cell. Commun. Signal.* **21**, 4 (2023). 36604720 10.1186/s12964-022-00859-7 PMC9814435

[37] de la Lopez, K. G., Toledo Guzman, M. E. & Sanchez, E. O. Garcia Carranca, mTORC1 as a regulator of mitochondrial functions and a therapeutic target in cancer. *Front. Oncol.* **9**, 1373 (2019). 31921637 10.3389/fonc.2019.01373 PMC6923780

[38] Szwed, A., Kim, E. & Jacinto, E. Regulation and metabolic functions of mTORC1 and mTORC2. *Physiol. Rev.* **101**, 1371–1426 (2021). 33599151 10.1152/physrev.00026.2020 PMC8424549

[39] Yaku, K., Okabe, K., Hikosaka, K. & Nakagawa, T. NAD metabolism in cancer therapeutics. *Front. Oncol.* **8**, 622 (2018). 30631755 10.3389/fonc.2018.00622 PMC6315198

[40] Arfin, S. et al. Oxidative stress in cancer cell metabolism. *Antioxid. (Basel)*. **10**, 642 (2021). 10.3390/antiox10050642 PMC8143540 33922139

[41] Chen, H. et al. Sirtuin 1 knockdown inhibits glioma cell proliferation and potentiates Temozolomide toxicity via facilitation of reactive oxygen species generation. *Oncol. Lett.* **17**, 5343–5350 (2019). 31186751 10.3892/ol.2019.10235 PMC6507466

[42] Mvunta, D. H. et al. SIRT1 regulates the chemoresistance and invasiveness of ovarian carcinoma cells. *Transl Oncol.* **10**, 621–631 (2017). 28667895 10.1016/j.tranon.2017.05.005 PMC5491457

[43] Xu, R. et al. Inhibition of NAMPT decreases cell growth and enhances susceptibility to oxidative stress. *Oncol. Rep.* **38**, 1767–1773 (2017). 28714034 10.3892/or.2017.5793

