# Supplementary material for: Targeting TRAF3IP2 disrupts cellular energetics through inhibition of NAMPT in triple negative breast cancer
Source: Sci Rep. 2025 Dec 23;16:133. doi: 10.1038/s41598-025-29057-4 (PMC12764808; doi:10.1038/s41598-025-29057-4)
Supplement: Supplementary file 1 — Supplementary Material 1 [file 41598_2025_29057_MOESM1_ESM.pptx]

## Slide 1
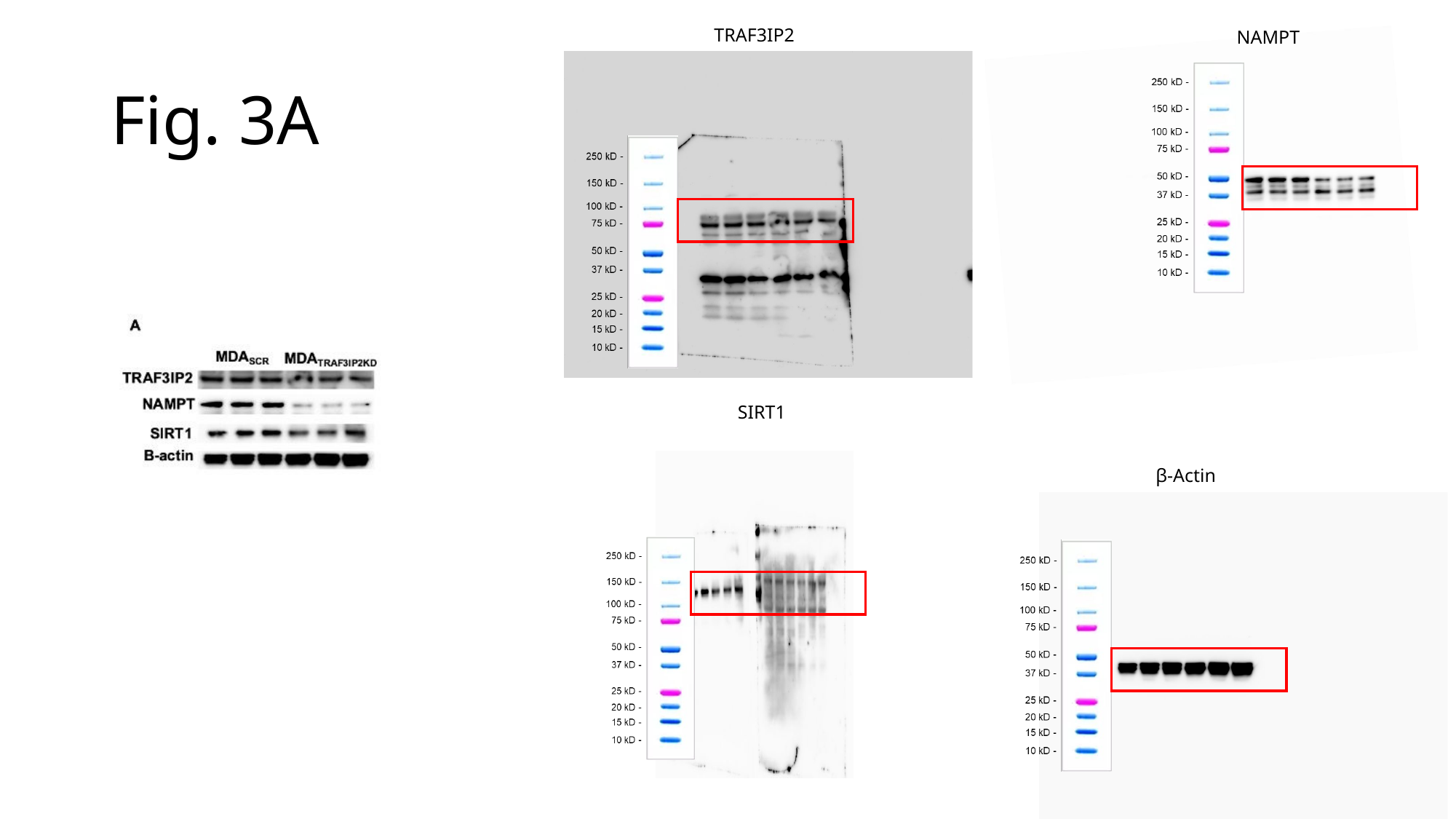

TRAF3IP2
NAMPT
# Fig. 3A
SIRT1
β-Actin

## Slide 2
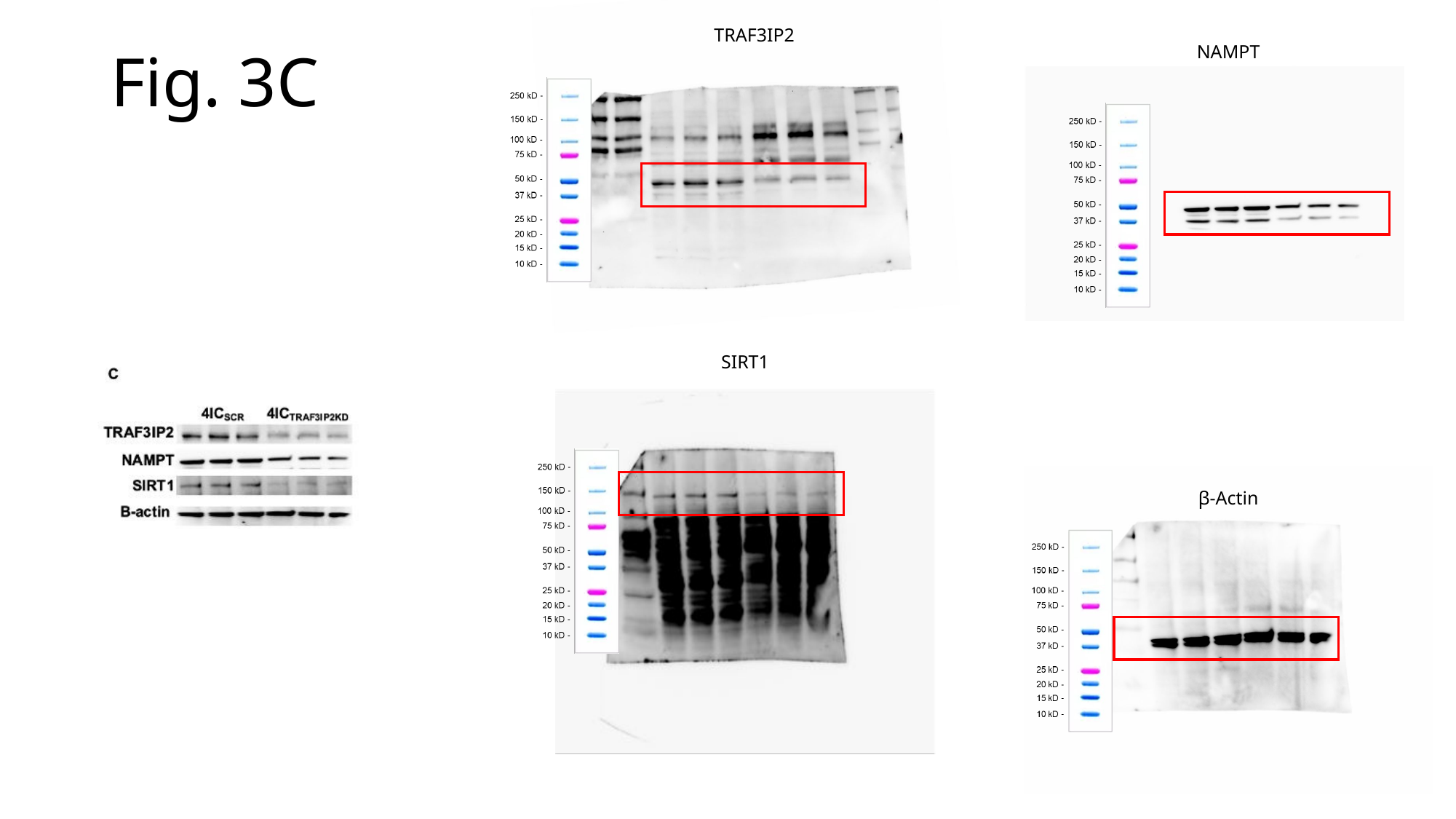

TRAF3IP2
NAMPT
Fig. 3C
SIRT1
β-Actin

## Slide 3
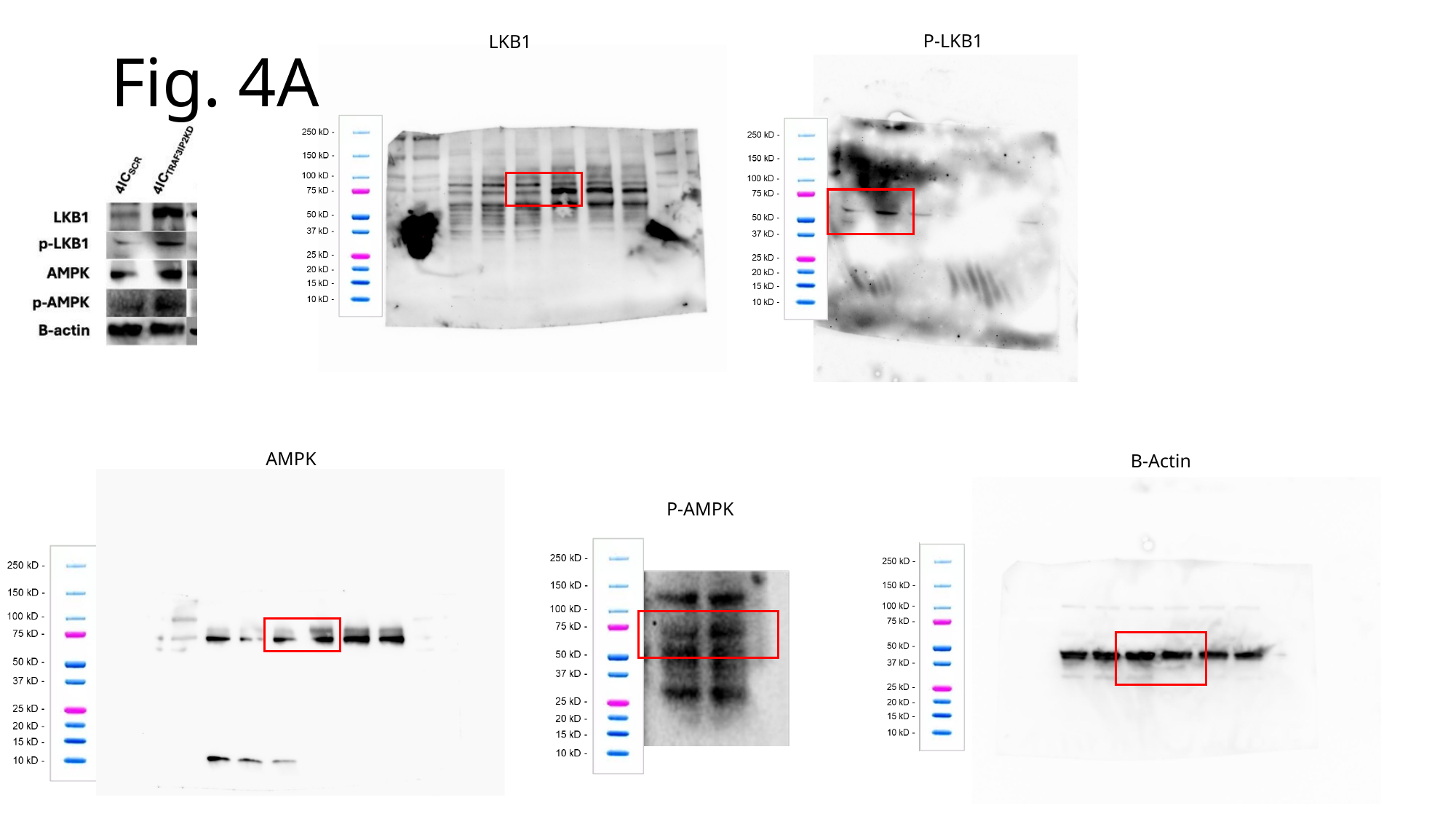

P-LKB1
LKB1
Fig. 4A
AMPK
Β-Actin
P-AMPK

## Slide 4
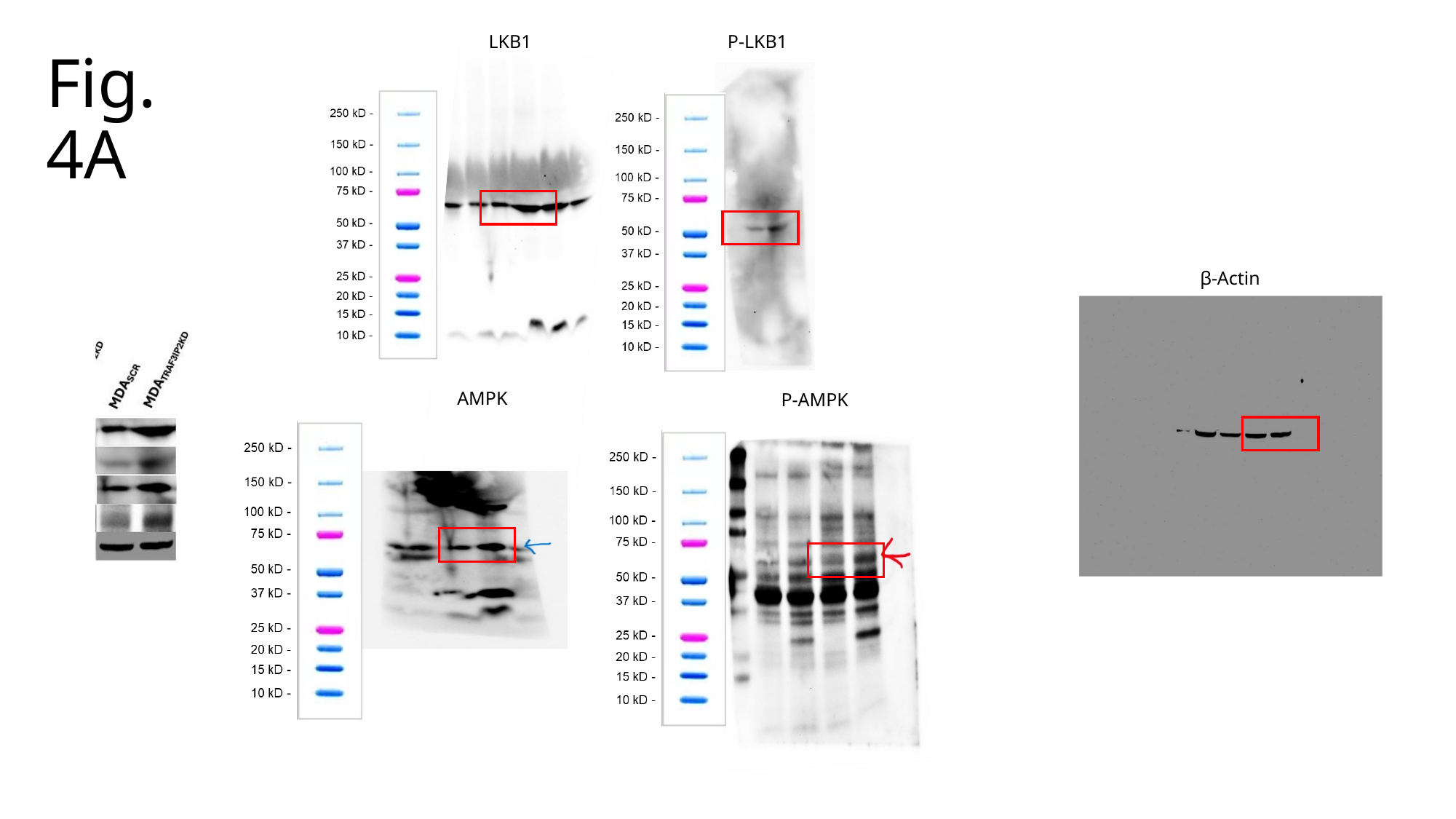

LKB1
P-LKB1
Fig. 4A
β-Actin
AMPK
P-AMPK

## Slide 5
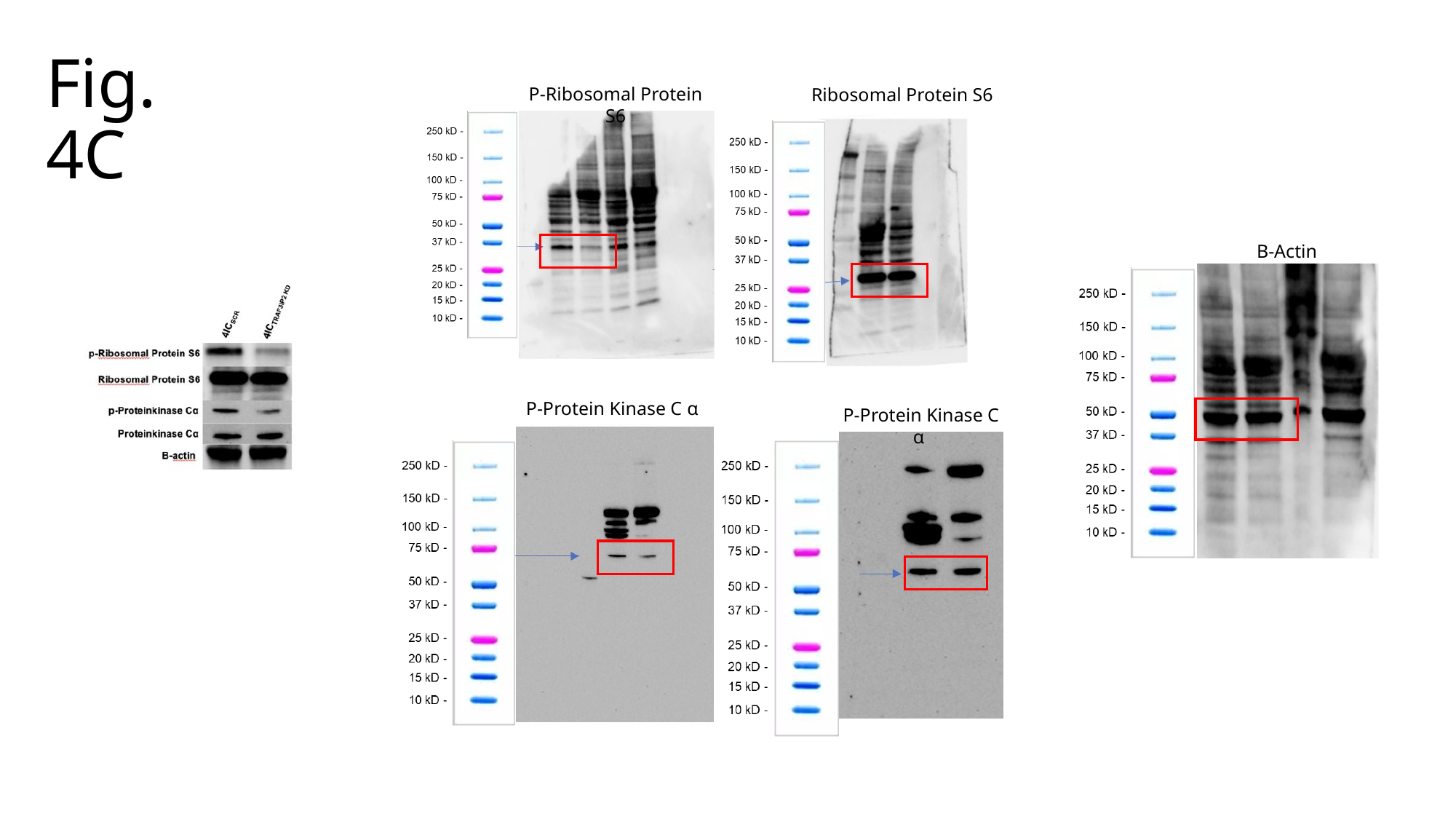

Fig. 4C
P-Ribosomal Protein S6
Ribosomal Protein S6
Β-Actin
P-Protein Kinase C α
P-Protein Kinase C α
